# Academic dishonesty by students of bioethics at a tertiary institution in Australia: an exploratory study

**DOI:** 10.1007/s40979-023-00124-5

**Published:** 2023-02-20

**Authors:** Jean Mukasa, Linda Stokes, Doreen Macherera Mukona

**Affiliations:** 1 Fatima College of Health Sciences, Ajman, United Arab Emirates; 2 Australia Catholic University, Melbourne Campus, Fitzroy, VIC Australia

**Keywords:** Academic integrity, Academic dishonesty, Plagiarism, Institutions of higher learning, Information ethics, Academic misconduct

## Abstract

**Background:**

Institutions of higher learning are persistently struggling with issues of academic dishonesty such as plagiarism, despite the availability of university policies and guidelines for upholding academic integrity.

**Methodology:**

This was a descriptive qualitative study conducted on 37 students of a Healthcare Ethics course at an Australian tertiary institution from February 2016 to October 2018. The purpose of the study was to explore the reasons for plagiarism detected the Turnitin^R^ plagiarism checking software and extensive review of manuscripts. The interviews were conducted in private rooms and in strict confidence. Thematic analysis was manually done.

**Results:**

Four major themes namely, lack of interest; pressure of time with competing priorities; lack of understanding of the policy on academic honesty, and “the determined students” were identified. Sub themes under lack of interest were lack of preparation and effort, low self-efficacy, poor studying techniques, and convenience of internet sources. Under pressure of time, the subthemes were, misplaced priorities, procrastination, high workloads, poor planning, competing interests, and the perception of availability of time at the start of the semester. Regarding lack of understanding of the policy on academic honesty, the subthemes identified were, lake of awareness of plagiarism, lack of awareness of acceptable similarity, conflicting messages from tutors and confusion with high school learning. The determined students were those that either made all effort to reduce plagiarism but still remained high, used the another language at home other than English, had poor paraphrasing techniques or lacked resources for English language editing.

**Conclusion:**

There are varied and diverse reasons for plagiarism. There is a need to systematically reinforce and educate students on issues pertaining to academic dishonesty and their associated implications.

## Introduction

Academic integrity is important for institutions of higher learning (De Maio et al. 2019). According to the International Centre of Academic Integrity (2021), academic integrity refers to a commitment to the six fundamental values of honesty, trust, fairness, respect, responsibility, and courage (International Centre of Academic Integrity, 2021). However, academic dishonesty has become a serious problem in institutions of higher learning. In academia, plagiarism, should have no place as it threatens the veracity of any discipline. It becomes even more serious in health professions education, where honesty and integrity are key (Ismail 2018; Lynch et al. 2017). Professional behaviors and attitudes are paramount in health professions education, because, if not properly reinforced, there is a risk of unprofessional behaviors in practice that can seriously compromise patient safety, and working relationships (Ismail 2018; Lynch et al. 2017). This is supported by findings from a recent systematic review that revealed plagiarism as a significant predictor of clinical misconduct (Fadlalmola et al. 2022). Studies conducted in different countries have reported high prevalence of plagiarism (Curtis and Tremayne 2021; Ewing et al. 2019; Hopp and Speil 2021; Ismail 2018; Ison 2015; Javaeed et al. 2019; Lynch et al. 2017).

The definition of plagiarism varies among scholars, but it refers to the use of an author’s words, ideas, reflections and thoughts without properly acknowledging them (Culwin and Lancaster 2001; Fishman 2009); with the aim of obtaining some benefit, credit, or gain which is not necessarily monetary (Fishman 2009; Tee and Curtis 2018). It is a form of cheating and theft of another person’s intellectual work (Koul et al. 2009). Gender, socialisation, efficiency gain, motivation for study, methodological uncertainties, easy access to electronic information and new technologies are reported in the literature as some factors influencing plagiarism (Jereb et al. 2018). A large survey conducted in Australia reported more factors associated with academic dishonesty that included lack of satisfaction with the teaching and learning environment, a perception of ‘lots of opportunities to cheat’, and not having English as a first language at home (Bretag, et al. 2019a, b). Fadlalmola and colleagues (2022), in a recent systematic review, reported a prevalence of 55.3% of plagiarism among nursing students. Another study conducted in Austria reported a prevalence of 22% among undergraduate students (Hopp and Speil 2021).

While plagiarism is still problematic in institutions of higher learning, there generally, has been a downward trend in its prevalence in the 30 years from 1990 to 2020 (Curtis 2022). The COVID 19 pandemic, might also have brought an increase in plagiarism and cheating by students (Comas-Forgas et al. 2021; Kratovil 2021; Wu et al. 2021). Similar concerns of cheating have been reported in the United States (cheating and plagiarism) (Perez-Pena 2012), Australia (Belot, 2016) and South Africa (News24, 2014). Plagiarism is also a problem among postgraduate students (Curtis and Tremayne 2021; Fatemi and Saito 2020; Kattan et al. 2017; Lines 2016b; Lynch et al. 2016; Selemani et al. 2018). It can come in the form of paraphrasing without referencing, or submitting another person’s work without proper acknowledgment among other forms (Fadlalmola et al. 2022; Muluk et al. 2021). It can also involve copying other people’s ideas and/or phrases without citing them, wrongly reporting direct statements, incorrectly interpreting information, or copying a full sentence structure but just changing the words without citing the owner of the work (Roman 2018). Academic dishonesty also includes deception, and fraudulent activity such as plagiarism, collusion, forging assessor signatures on practice assessments, or cheating in exams (Tee and Curtis 2018). Some students resort to the use of cheat sheets, direct copying from colleagues during written tests, plagiarism, and falsification of data (Comas et al. 2011) as well as hiring external contacts who secretly communicate answers to them during examinations (Lancaster et al. 2019).

One of the major drivers reported, has been the easy access to information on the internet (De Maio et al. 2019; Ison 2015, 2018; Muangprathub et al. 2021). Moreover, the massification and internationalisation of higher education have resulted in the recruitment of large and diverse student cohorts, unfortunately, without corresponding growth in institutional funding (Bretag et al. 2019a, b). This has resulted in most universities operating as commercial enterprises, with all operations being driven by competitive strategies. This might have the undesirable effect of reduced student supervision, which is a gateway for academic dishonesty. In addition, a plethora of resources have emerged that provide sharing of resources (De Maio et al. 2019; Gee 2018; Ison 2015), and this has created a booming ‘sharing economy’ and has enabled dissemination of academic information via online platforms. This has allowed students to outsource important tasks, contract cheating, making academic success more of what one can access rather than what one knows (Richardson 2015). Ellery (2008) argues that there sometimes is no deliberate intent to plagiarise. In such cases it is most probably due to poor referencing norms, poor writing skills and lack of real engagement with plagiarism and referencing issues (Ellery 2008). This can be true among international students, with their proficiency barriers as non-native speakers (Bretag et al. 2019a, b). Yet still, some authors have implicated personality traits in cheating tendencies. According to Wilks et al. (2016), inclination to plagiarize is negatively associated with Conscientiousness and Agreeableness traits (Wilks et al. 2016). It is also positively corelated with external stress ad pride (Romanowski 2022). Some students simply fail to grasp the concept of plagiarism (Breen and Maassen 2005).

To date, the Australian media has reported a considerable number of cheating scandals among students in higher education (Chung 2015; De Maio et al. 2019; Perez-Pena 2012; Visentin 2015). Unlimited exposure to information and communications technology (ICT) in homes and educational institutions has been implicated (Considine et al. 2009; Evering and Moorman 2012; Ferraro 2018; Gee 2018). Moreover, publications in ICT media are usually available to the public, and issues of the intellectual property of the authors are not pertinent as ownership of one’s work is relinquished at the time of publication (De Maio et al. 2019; Evering and Moorman 2012). Typically, students can use gadgets such as smart phones and tablets for e-learning through their learning platforms wherever there is internet access (Anshari et al. 2017; Kibona and Rugina 2015), which is the case in most Australian universities. This makes access to information that is online very easy at any given time, and this renders the students vulnerable to academic dishonesty.

We conducted our study at a university situated in the State of Victoria, Australia, that trains students of health sciences, and humanities. Unfortunately, estimating the actual prevalence of plagiarism is difficult because perpetrators will normally not freely admit to it (Hopp and Speil 2021). Ordinary interviews or even anonymised questionnaires might not yield data that reflects the actual prevalence of plagiarism in an academic institution. For these reasons, the students in our study were, therefore, identified during routine assessment for originality of a Healthcare Ethics course assignment at the Australian tertiary institution from 2016 to 2018. The course is a core unit therefore is compulsory for students from Nursing, Midwifery, Paramedicine, Biosciences and Allied Health disciplines. The purpose of this qualitative retrospective review was to explore the reasons for plagiarism among these students taking the Healthcare Ethics course.

## Methodology

This was a descriptive qualitative study conducted on 37 students of a Healthcare Ethics course at an Australian tertiary institution between 2016 and 2018. The purpose of the study was to explore the reasons for plagiarism among students of a HealthCare Ethics Course. Students were identified during routine assessment for originality, by the Turnitin^R^ plagiarism checking software. This was a written assignment in the Healthcare Ethics course of 2500 words. Students could pre-emptively review their drafts for text-matches and could make all the necessary adjustments to bring the level of similarity down. They were given 6 weeks to prepare and submit the assignment.

Of all the 523 students who wrote the assignment, 179 records, corresponding to 179 students had a similarity of above 15%. Out of these 179 records with high similarity, data saturation for the qualitative review was reached at 37 records, corresponding to 37 students. Data were collected over 2 academic years from February 2016 to October 2018. The group comprised second and third year students undertaking undergraduate degrees in Nursing, Midwifery, Paramedicine, Biosciences, Physiotherapy, Occupational Therapy and the Enrolled Nurse Diploma Program. Documents with a similarity above 15%, which is the cutoff acceptable at the university, were identified. Faculty then extensively reviewed the associated transcripts from Turnitin^R^ to ensure that the similarities were not due to common headings and references. The students with a high similarity, due to copying and pasting phrases directly from the internet, copying from previous similar assignments, and not referencing at all, were contacted by e-mail and requested to attend an interview. The interviews were done by 2 staff members who also took detailed notes. The interviews followed a semi structured questionnaire that had questions addressing whether the students were aware that they had plagiarized, whether they were aware of the university’s plagiarism policies, the reasons for the plagiarism and whether they were aware of the consequences. The interviews were conducted in private rooms and confidentiality was observed. Though the data used for this study were collected during routine disciplinary proceedings, the students gave verbal consent for use of the findings both in this study and for use in routine assessment, provided their identities remained anonymous. The approval for the study was granted by the department’s ethics committee. Thematic analysis, as recommended by Clarke et al. (2015) was manually applied to the data following the stages of data organization, familiarization, transcription, coding, application of a thematic code, displaying and reporting (Clarke et al. 2015). Trustworthiness was ensured by observing credibility, transferability, dependability, and confirmability (Lincoln and Guba 1986). We spent a considerable amount of time with each student as we explored their reasons for academic dishonesty (prolonged engagement). There was also a thick description of the events in the generated transcripts to ensure transferability of findings.

## Findings

This section presents the findings of the study. Table 1 displays distribution of the students according to various disciplines. There was a total of 179 students interviewed over a period of 2 academic years from February, 2016 to October, 2018. However, only 37 records, after data saturation, were included in the final analysis.

**Table 1:** Demographic Data (*n* = 37)

Variable	Number of students	Percentage(%)
**Age (Years)**		
**18–25**	24	64.8
**26–30**	9	23.5
**> 30**	4	11.7
**Gender**		
**Male**	9	23.5
**Female**	28	76.5
**Level of training**		
**Year 2**	19	51.4
**Year 3**	18	48.6
**Citizenship**		
**Local**	26	69.8
**International**	11	30.2
**Discipline (Bachelor’s Degree)**		
**Physiotherapy**	2	4.5
**Occupational Therapy**	1	2.8
**Public Health**	1	3.4
**Biosciences**	3	7.3
**Midwifery**	6	15.6
**Paramedicine**	8	22.3
**Nursing**	13	35.2
**Nursing Enrolled Nurse Diploma of Nursing Entry**	3	8.9
**TOTAL**	**37**	**100.0**

Table 2 presents the themes and subthemes identified in the interviews. All the recorded data from the interviews conducted with the students regarding academic dishonesty were analysed and four predominant themes emerged. These were: lack of interest; pressure of time with competing priorities; lack of understanding of the policy on academic honesty, and “the determined students”.

**Table 2:** Themes and subthemes

Theme	Subthemes
**Lack of interest or effort into the assignment**	Lack of preparation
	Lack of effort
	Low self-efficacy
	Poor study techniques
	Convenience of internet sources
**Pressure of time with competing priorities**	Misplaced priorities
	Competing interests
	Procrastination
	High workloads
	Poor planning
	Perception of availability of time at the start of the semester
	Poor time management
**Lack of understanding of the policy on academic honesty**	Lack of awareness of plagiarism
	Lack of awareness of acceptable similarity
	Conflicting messages from tutors
	No experience with referencing
	Confusion with high school learning
	Lack of insight about plagiarism
**“The determined students”**	All effort made to reduce plagiarism but remained high
	Use of another language other than English
	Poor paraphrasing techniques
	Lack of resources for English Language editing

### Theme 1: Lack of interest or effort into the assignment

Most students interviewed demonstrated lack of interest in the course. Despite multiple avenues explaining the expectations of the assignment, students indicated that they were not prepared for the assessment and did not think they could master it. All the students, both second and third year, in our study had received training in academic writing. Some students demonstrated lack of effort put into the submission and simply rode on the convenience of internet sources. This theme was highlighted by the students stating:

*I thought you were only going to mark the oral presentation and not the scripts.* (Year 3, Occupational Therapy)

*I wish you had told us about the expectations of the assessment. That would have prepared us better. I did not think it was really feasible to put references for everything.* (Year 2, Nursing)

### Theme 2: Pressure of time with competing priorities

This theme was echoed by many students interviewed. Students cited competing interests, and high workloads which were generally due to poor planning, misplaced priorities, procrastination, and perception of the availability of time at the start of the semester. These were second- and third-year students who were very much aware of the workloads in the ethics courses and should have set time aside for those. Some of the students had this to say:

*I have so much going on in my life. I didn’t have much time to write the assignment.* (Year 2, Nursing)

*There is so much required. I can’t possibly manage. I think you could be more lenient.* (Year 3, Nursing)

*I run out of time.* (Year 2, Paramedicine)

### Theme 3: Lack of understanding of the policy on academic honesty

Many students demonstrated lack of understanding of the policy on academic dishonesty. Some were not aware of plagiarism itself while some simply confused university with high school learning where they could take statements straight out of textbooks. Other students cited receiving confusing messages from tutors. This theme is reflected in the following excerpts:

*I didn’t think 27% was bad. I thought anything less than 30% was okay. I learnt in my first year, in another unit, that I can cut and paste the scenario at the top of my essay. Obviously, this format doesn’t reflect the expectations of all tutors which I now know and won’t be doing again in the future.* (Year 3, Physiotherapy)

*This is awful. No one has ever talked to me about this before. I always submit my assignments, and no one told me I was doing anything wrong. I am shocked to find out now after so many submissions before.* (Year 2, Nursing)

*I have never done academic references before. I don’t think I will be any good at it.* (Year 3, Public Health)

### Theme 4: “The determined students”

There were some students who were aware of the concept of plagiarism and made all effort to bring their similarity down to the acceptable levels. However, they failed because they lacked good paraphrasing techniques owing to usage of English as a second language. Most of these students also lacked knowledge of how to access professional English language editing services provided by the institutions’ academic support services. Some of these students had this to say:

*I have been working on getting my similarity down on my assessment all day and no matter what I do it is still sitting at 30%. I have referenced everything so I’m unsure why this is happening.* (Year 3, Biosciences)

*What does my similarity percentage have to be under and is this okay? I’m not sure what else to do.* (Year 3, Midwifery)

## Discussion

Four major themes were identified in our study. These were “lack of interest or effort into the assignment”, “pressure of time with competing interests”, “lack of understanding of the policy on academic dishonesty” and “the determined students”. These themes are discussed in detail in this section.

### Lack of interest

One theme that was identified in our study was “lack of interest”. Some students reported not having prepared for the assignment, lacking effort, low self-efficacy and poor study techniques. Fatima et al. (2020) have also reported low self-efficacy in addition to poor training and inadequate writing as drivers of plagiarism in a study conducted in Pakistan (Fatima et al. 2020). Some students in our study confessed the convenience of internet sources. In agreement to this, Muluk et al. (2021) reported ease of access to online material as a reason for plagiarism in a study conducted in Indonesia (Muluk et al. 2021). However, they just copied information without an effort to understand it and paraphrase. This has been widely reported in literature with one report by Gullifer and Tyson (2014) coming from Australia. According to Ismail (2018) and Skaar and Hammer (2013) one common driver for academic dishonesty is the lack of interest in an assessment task allocated on the part of the student (Ismail 2018; Skaar and Hammer 2013). Findings of a study conducted in Australia revealed that almost half of the students interviewed did not bother to read the university plagiarism policies (Gullifer and Tyson 2014). Similarly, a study conducted by Yu et al. (2017) reported that, lack of self-control, others-oriented life purpose, lack of academic preparation, and extracurricular activities involvement were significantly associated with academic cheating. The attitude of the students towards academic dishonesty, lack of punitive measures from faculty and the availability of an enabling environment for cheating may also perpetuate this problem (Eaton et al. 2020; Yu et al. 2017). In support of this notion, Brimble (2016) asserts that students not challenged for plagiarizing in their submissions tend to cultivate a sense of confidence to continue to transcribe in subsequent assessment tasks (Brimble 2016). The past achievements that came from plagiarizing, too often motivate the student into believing that it was worth the risk (Tee and Curtis 2018). The gains from transcriptions out-weigh the risks of being caught, so the driver becomes personal and the student continues at their own peril (Lancaster and Clarke 2016). As a result, they take short cuts so that they finish their tasks quickly in order to pursue personal interests. Such students are content to cut and paste from published sources with little regard for referencing formalities and pass this work off as their own (McCabe et al. 2012; Skaar and Hammer 2013).

As some students progress with their education, they shift from plagiarism to contract cheating or employing ghost writers due to the increasing abundance and accessibility of websites (Walker and Townley 2012). Advertising is linked to students’ internet sites and guarantee custom written, personalized papers that do not contain plagiarism. Websites promote affordable papers to entice the student to make contact (Lancaster and Clarke 2014; Walker and Townley 2012). When students have already plagiarized and lacked the drive to learn, the temptation to use a ghost writer is not a difficult choice (Lancaster and Clarke 2014). Furthermore, the more technically savvy millennials have learnt the shortfalls within the text matching systems such as Turnitin^R^ and find ways to work around having their submissions identified as plagiarized (Skaar and Hammer 2013).

Additionally, easy access to electronic information and services via the internet such as essay banks and ghost-writing websites, coupled with their persuasive marketing techniques and active targeting tactics, encourage students’ involvement and enhance their vulnerability (Ison 2018; Newton, 2018; Rowland et al. 2018). However, the more movement of data and the more authors students have access to, the greater the chance they have of losing sight of who has the intellectual property that students may claim to be their own (Šprajc et al., 2017); rendering them vulnerable to plagiarism. Limited or non-existent support challenges students to weigh up the risks of the consequences of transcribing and make a moral decision. The student will rationalize why it is acceptable to plagiarize which helps them to justify or neutralize their actions (Brimble 2016). Justification will only appease their actions as they will know that this is still wrong (Beasley, 2014). However, Dezcallar et al. (2014) argue that students become more focused and less likely to cheat as they reach their final year of undergraduate studies (Dezcallar et al. 2014).

### Pressure of time with competing priorities

Another theme that was identified from our study was pressure of time with competing priorities. Some students bemoaned high workloads, procrastination and competing interests. Many authors have reported similar findings and according to them, some students are poor time managers and some authors have reported a significant association between procrastination and cheating among students of higher learning (Dezcallar et al. 2014; Muluk et al. 2021; Rigby et al. 2015; Yu et al. 2017). The authors mentioned here state that grades of the procrastinating student in their first year of university are generally poor; as they fail to balance life and study (Dezcallar et al. 2014; Rigby et al. 2015; Yu et al. 2017). As semesters come to an end, the rush to complete essays and study for exams places them under considerable pressure. Students go into damage control as a means of survival to meet the contending priorities. A student distracted by commitments beyond their studies such as sport, work or family has less time to focus on studies and use this as a driver to turn to academic dishonesty (McCabe et al. 2012). This was the case with some of the students in our study who claimed that they had so much happening in their lives. These included sport, part time jobs and family issues especially among females. The importance of setting priorities was emphasized to these students.

Lines (2016a, b) found that contract cheating websites have often portrayed students as hard working and determined yet struggling; and that their work life balance was suffering. The author continues to argue that the commercialization of universities has also resulted in high student numbers in tutorials and a lack of personalization resulting in feelings of being neglected among students. Websites promotional advertising will empathise with a student’s lack of support to justify their purchase and assist them in their learning process. For the student that lacks confidence in their own abilities, the purchasing of a paper has considerable appeal. For others the transcription is a convenience and time saver (Lines 2016a).

### Lack of understanding of the policy on academic honesty

Some students in our study reported lack of awareness of the policies on academic honesty. This was unexpected because all students go through an academic writing course in their first year. They are given information on plagiarism and other aspects of academic dishonesty. Above that, they also go through library orientation where they are taught about academic dishonesty, its implications, and its penalties. This is similar to findings of a study conducted in Iran by Zarfsaz and Ahmadi (2017), where students claimed having inadequate information about how not to plagiarize despite their awareness of the concept and definition of plagiarism (Zarfsaz and Ahmadi 2017) and another conducted in Indonesia (Muluk et al. 2021). A study by Gullifer and Tyson (2014) conducted to assess numbers of students reading plagiarism policies revealed that only about half (52%), of the 3,405 surveyed had actually read the universities’ policies (Gullifer and Tyson 2014). Gullifer and Tyson (2014) conducted their study in Australia, so their findings are very relevant to the context of our present study. They might confirm the reluctance of students to read and understand plagiarism policies. All the students interviewed in our study reported awareness of the institution’s plagiarism policy. However, some of them reported not having read the documents in detail. Another study conducted in Iran reported that more than a third (34.8%) of the participants did not know what plagiarism was while 72% were not aware of the legal consequences of plagiarism (Ismail 2018). Similarly, findings from another study conducted in Nigeria among postgraduate students revealed inadequate writing skills and lack of knowledge of what constitutes plagiarism, contributed to plagiarism (Idiegbeyan-Ose et al. 2016).

According to Evering and Moorman, students at tertiary level are frequently challenged with more advanced assessment topics and are expected to apply, analyse and evaluate to demonstrate knowledge (Evering and Moorman 2012). This includes interpreting publications into their own words and applying them to their assessment task. Restating the essence of a publication can be straight forward when tasks and publications are simple, but students may struggle to rework content with more complex topics. Consequently, they will relent and transcribe or cut and paste, which moves information around quickly and minimises pressure.

### “The determined students”

There was one group of participants that we termed “the determined students”. These are the students who unintentially committed plagiarism due to poor paraphrasing techniques, use of another language at home other than English and lack of resources for English language editing. These students tend to be under some form of pressure, to be successful in life and to apease their parents, and to finish their studies (Pérez-Peña 2012). About a third (30.2%) of our participants were international students. Some of them did not speak English as their first language at home. Their plagiarism noted was mainly due to lack of paraphrasing and proper attribution of work to the rightful authors. According to Rowland and colleagues (2018), these students do not set out to cheat, but may be persuaded to do so in order to resolve their impasse (Rowland et al. 2018). In support, Bretag and colleagues assert that these students become vulnerable to dishonest behavior as they grapple with the English language (Bretag et al. 2019a, b; Zarfsaz and Ahmadi 2017). This was observed in our study whereby some students were copying whole paragraphs of information from the internet and pasting it into their work. Though properly referenced, one should paraphrase. Some students mentioned lack of resources for language editing. According to Bretag et al. (2019a, b), international students often experience financial strain and need to work during the semester (Bretag et al. 2019a, b). Less command over English language was cited as the main reason of plagiarism in a study conducted in Iran (Zarfsaz and Ahmadi 2017). Idiegbeyan-Ose and colleagues, in a study conducted in Nigeria also reported inadequate writing skills and lack of knowledge of what constitutes plagiarism as drivers of plagiarism among students (Idiegbeyan-Ose et al. 2016). Due to the cost of education and the need to avoid repeating units, students might turn to dishonest practices to get a pass (Bretag et al. 2019a, b). International students at our university are required to pay their own fees and failing units translates to more cost for their education. Some of them took odd jobs to raise more income and this took a lot of time off their studying. Research has shown that students on employment are more likely to have divided attention between work and studies (Romanowski 2022). Juggling employment to meet financial needs and the extra time needed to interpret the meaning and write an assessment task can place students under considerable stress.

According to Bretag et al. (2019a, b), when language barriers create isolation for English as second language students (ESL), coupled with large tutorial class numbers, the prospect of employing a ghost writer is more appealing (Bretag et al. 2019a, b). Empowered by the perceived lack of support, students assess their options. International and ESL students may take longer to read and digest the meaning of an article, faltering in the language and then struggle again to rework this into their own words. Particularly when they have a limited vocabulary, think and process meaning in their native language then covert it back to academic English (Zigunovas 2017). While native English-speaking students have more time to master paraphrasing and writing skills, ESL students must fall back on their local habits and often revert to copying from texts with limited vocabulary at the start of their education. In effect international students do not cheat any more than local students (Quaye et al. 2019). As with many native English-speaking students, ESL students struggle with writing with the hidden issues of academic dishonesty emanating from cultural values (Husain et al. 2017). A study conducted in Australia reported inadequate and insufficient support programmes for international students. There is no sufficient time with students to teach them proper ways of referencing. Above this, ESL students might also have time limitations due to poor time management skills (Muluk et al. 2021). Though some authors argue that a high proficiency in English writing skills is not protective against unintentional plagiarism among international students in a new academic settings (Fatemi and Saito 2020; Bretag et al. 2019) insist that language barrier, rather than culture, is a more important reason for cheating among international students in Australia (Bretag et al. 2019a, b). This underscores the importance of adequate support for these students (Tee and Curtis 2018).

The students in our study came from 8 different health care programs and they were all taking the Health Care Ethics course. The majority of them (76.5%), were females and most of them (64.8%), were aged between 18 and 25 years. The finding that there were more females who were dishonest than males could be related to the fact that there is generally less male than female enrollment into nursing programs in Australia. In 2017, registration data for Australia showed that men made up only 11.75% of the registered nursing workforce (Nursing and Midwifery Board of Australia, 2017). Similarly low proportions have also been observed in other similar countries such as the United Kingdom (10.2%), the United States of America (7.2%) and Canada (6%) (Stanley et al. 2014). This ratio is evident in the participants cohort of this study where there were significantly more female students. However, it was noted that the ratio of males to females was similar in other disciplines at the time. Some authors have reported significant associations between dishonest behavior and gender, socioeconomic status, and level of training (Yu et al. 2017). McCabe et al. have reported that male students cheat more than female students (McCabe et al. 2012). In agreement, Ismail, in a study conducted in Iran reported a prevalence of plagiarism of 54.3% and it was significantly higher among male students (Ismail 2018). Other authors have reported a negative association between plagiarism with age, parenting, and completing semester credits and a positive correlation between plagiarism with average grades and liberal educators (Fadlalmola et al. 2022). Although one author has reported decreasing incidence of plagiarism with academic level (Romanowski 2022), it was also noted in our study that there were almost equal proportions of year 2 and year 3 students in our participants. However, our study being qualitative, no associations could be inferred from the results. Further quantitative studies, should be conducted at the university to have more insight in academic dishonesty issues in terms of associated factors and strategies to curb it.

Almost a third (30.2%) of the cohort were international students. Learning about the cultural ways and nuances of their new home takes time and cannot always be explained in a way that is understood (McKinstry et al. 2020). Students may feel overloaded with instructions at orientation at the start of the semester and are unable to retain and consolidate large quantities of information. Some cultures, such as the Asian, place great value on the grades achieved as a demonstration of success (Skaar and Hammer 2013) and there is a general belief that higher degrees bring higher paying jobs and are prestigious (Pérez-Peña 2012). Perez-Pena (2012), also reported pressure to have good grades from parents, in a study conducted in the United States of America. As a result, higher qualifications are the goal and placed above knowledge and skill (Ison 2018; Skaar and Hammer 2013). Some students felt pressure from parents to excel and bring honour to the family, and better employment prospects for themselves. Parents make clear to their young, the sacrifices made to get them to foreign/ western countries to achieve their qualifications, a burden these foreign students carry with them. Once the child leaves home, the driving force is no longer pushing them to succeed, and they must then rely on their intrinsic drive.

## Conclusion

Academic dishonesty was high among the students at our institution. There was a wide range of reasons why students resort to plagiarism. However, no matter the reasons, plagiarism is unacceptable in academia and stern measures should be taken to curb it. In some institutions it is dealt with in informal ways without reporting and this, unfortunately, is not an effective deterrent measure (Eaton et al. 2020). Ferraro (2018) argues that despite all the highlighted issues with plagiarism, students are made aware, on entry to tertiary institutions, of the importance of academic honesty and integrity. Though students are given orientation regarding the use of library resources that typically includes accessing resources, writing skills, plagiarism and platforms for interaction, not all students deem reading plagiarism necessary. It is imperative to systematically address this challenge especially among nurses and other students of health professions. It is also very important to give relevant support to international students regarding English language proficiency and other issues that compromise their academic integrity. It is argued that, demographically diverse settings, acquiring values, attitudes, norms, beliefs, and practices that help prevent plagiarism must be a long-term and iterative process (Ellery 2008). Health care is a field where honesty and integrity are key and unethical academic conduct will most likely translate into unethical practice and undesirable professional behavior (Tee and Curtis 2018; Theart and Smit 2012). Lecturers and tutors should continuously relay messages regarding plagiarism to students (Gullifer and Tyson 2014; Tee and Curtis 2018). Innovation is very essential in addressing plagiarism. Approaches such as the “3-step approach” (Ng and Yip 2019), “Plagiarism warfare” (Ade-Ibijola et al. 2022) and the “Goblin Threat” (Kier 2019) have been shown to improve awareness of plagiarism. However, the students in our study were reminded of the unacceptability of plagiarism when the assignment was given.

Our study had its limitations. Data for this study was collected routinely as students submitted their assignments, however students gave their verbal consent for future use of the findings. Important cues such as non-verbal might have been overlooked during data collection because at the time, the major aim was disciplinary action for the students. The researchers, however, took detailed notes as the students narrated their stories. Future work regarding academic dishonesty should also look at how the COVID-19 pandemic has impacted academic integrity. There is also need to conduct quantitative, multicentre, longitudinal studies to examine the dynamics of plagiarism as students progress with their studies and the factors associated with it.

## Data Availability

All data is available from the authors on request.
